# Identification of Genetic Risk Factors for Neonatal Hyperbilirubinemia in Fujian Province, Southeastern China: A Case-Control Study

**DOI:** 10.1155/2018/7803175

**Published:** 2018-09-12

**Authors:** Jinfu Zhou, Changyi Yang, Wenbin Zhu, Shuwei Chen, Yinglin Zeng, Jing Wang, Hong Zhao, Yao Chen, Feng Lin

**Affiliations:** ^1^Center of Neonatal Screening, Fujian Provincial Maternity and Children's Hospital, Affiliated Hospital of Fujian Medical University, Fuzhou 350001, Fujian Province, China; ^2^Department of Neonatology, Fujian Provincial Maternity and Children's Hospital, Affiliated Hospital of Fujian Medical University, Fuzhou 350001, Fujian Province, China

## Abstract

To date, the genetic risk factors for neonatal hyperbilirubinemia remain unknown in Southeastern China. This case-control study aimed to identify the genetic risk factors for neonatal hyperbilirubinemia in Fujian, Southeastern China. A total of 286 hyperbilirubinemic newborns were enrolled as a case group, and 250 randomly selected newborns without jaundice or with a bilirubin level that was lower than the threshold required for phototherapy served as controls. The serum levels of total bilirubin, unconjugated bilirubin, and direct bilirubin were measured, and the common genetic loci in UGT1A1, OATP1B1, and HO-1 genes were genotyped. Higher incidence of ABO incompatibility and G6PD deficiency was detected in the case group compared to the control group (*P* < 0.01). There were significant differences in the frequencies of rs4148323 and rs1805173 genotypes between the case and control groups (*P* < 0.05). At the rs4148323 locus, the frequencies of GA heterozygotes and AA mutant homozygotes were higher in the case group than in the control group (*P* < 0.05), and at the rs1805173 locus, the frequencies of LS, MS, and SS genotypes were higher in the case group than in the control group (*P* < 0.05). A higher frequency of rs4148323 A allele and rs1805173 S allele was detected in the case group compared to the control group (*P* = 0). Additionally, multivariate logistic regression analysis identified that the mutant genotype of rs4148323 in the UGT1A1 gene, ABO incompatibility, G6PD deficiency, and SS genotype at rs1805173 locus of the HO-1 gene were genetic risk factors of neonatal hyperbilirubinemia. Our data demonstrate that G211 mutation in the UGT1A1 gene, ABO incompatibility, G6PD deficiency, and the SS genotype of the repeats in the promoter region of the HO-1 gene are risk factors for neonatal hyperbilirubinemia in Fujian, Southeastern China.

## 1. Introduction

Hyperbilirubinemia is a common disorder in newborns [1, 2]. Severe hyperbilirubinemia may cause bilirubin encephalopathy, and even the survivors may develop chronic or permanent damage and sequelae in the nervous system [3–5]. Although the exact pathogenesis of neonatal hyperbilirubinemia has not been fully understood, perinatal and genetic factors are widely accepted to jointly contribute to this disorder [6–8].

As an inducible enzyme and rate-limiting enzyme of heme degradation, heme oxygenase-1 (HO-1) may decompose heme to biliverdin, which is subsequently reduced to unconjugated bilirubin (UCB) [9]. Previous studies have shown the anti-inflammatory, antioxidant, and antiapoptotic activities of HO-1 [10]. It has been shown that the short (GT)_n_ allele in the promoter region of the HO-1 gene may affect HO-1 transcription, and the number of (GT)_n_ alleles negatively correlates to HO-1 transcription, while short (GT)_n_ repeats may cause an increase in the HO-1 transcription, resulting in enhancement of heme catabolism and an increase in the heme production [9]. Uridine diphosphate glucuronosyl transferase 1A1 (UGT1A1) is an important metabolic enzyme in the bilirubin metabolism pathway [11]. Under UGT1A1 catalysis, UCB reacts with uridine diphosphate glucuronate to form direct bilirubin (DBIL), which is easy to be excreted via bile due to an increase in the water solubility [11]. Organic anion transporting polypeptide 1B1 (OATP1B1), a transport protein that is specifically located in the hepatocyte basolateral membrane, absorbs UCB to liver, thereby participating in UCB binding and metabolism [12].

In addition to glucose-6-phosphate dehydrogenase (G6PD) deficiency and ABO incompatibility, the associations of genetic polymorphisms in the three bilirubin metabolism genes, including UGT1A1, OATP1B1, and HO-1, with the susceptibility to hyperbilirubinemia, have been extensively examined [13–16]; however, the associations of the polymorphisms in the same gene locus with the development of hyperbilirubinemia vary in the study areas and populations [17–20]. To the best of our knowledge, there is no knowledge on the genetic risk factors for neonatal hyperbilirubinemia in Fujian Province, Southeastern China. This case-control study was therefore designed with aims to identify the genetic risk factors for neonatal hyperbilirubinemia in Southeastern Chinese populations.

## 2. Methods

### 2.1. Ethical Statement

This study was approved by the Ethics Review Committee of Fujian Provincial Maternity and Children's Hospital (permission no. 2015-077). Signed informed consent was obtained from all participants' guardians following a detailed description of the purpose of the study.

### 2.2. Study Subjects

A total of 286 hyperbilirubinemic newborns admitted to the Department of Neonatology, Fujian Provincial Maternity and Children's Hospital (Fuzhou, China), during the period between May 2015 and September 2016 were enrolled in this study, and 250 randomly selected newborns without jaundice or with a bilirubin level that was lower than the threshold required for phototherapy in the hospital during the study period served as controls. The newborns with a body weight of < 2000 g, < 35 weeks gestational age, severe infections, congenital biliary atresia, positive hepatitis B surface antigen (HBsAg), or other birth defects were excluded from the study. All subjects were Chinese individuals without genetic relationships, and the gender, body weight, gestational age, type of delivery, and type of feeding were collected from the subjects' medical records.

### 2.3. Diagnosis of ABO Compatibility and G6PD Deficiency

ABO compatibility was defined as an O blood type for mothers and either A or B blood type for children [21].

For the diagnosis of G6PD deficiency, heel capillary blood samples were collected from the subjects and used for measuring the G6PD activity. Those with a G6PD activity of < 2.6 U/g hemoglobin were further subjected to quantitative G6PD enzymatic assay using the G6PD nitroblue tetrazolium (NBT) Quantification Ratio Kit (Micky; Guangzhou, China). A G6PD/6-phosphogluconate dehydrogenase (6PGD) ratio of ≤ 1.0 was defined as a G6PD deficiency [22].

### 2.4. Measurement of Bilirubin Levels

Fasting blood samples were collected from the subjects, and the serum levels of total bilirubin (TBIL), UCB, and DBIL were measured with a fully automatic biochemical analyzer (Abbott Laboratories; North Chicago, IL, USA). Hyperbilirubinemia was defined as any serum TBIL concentration of >95th percentile for age in hours according to the hour of life-specific bilirubin nomogram [23].

### 2.5. Genotyping of Genetic Variants

Venous blood samples (2 to 3 ml) were collected and transferred to EDTA-anticoagulated tubes (Fuzhou Changgeng Medical Devices Co., Ltd.; Fuzhou, China). Genomic DNA was extracted from EDTA-anticoagulated blood samples using the High-pure Genomic DNA Isolation Kit (Guangzhou Hers Biotechnology Co., Ltd.; Guangzhou, China) and stored at −20°C for the subsequent experiments.

The loci of rs4148323 (c.G211A, p.Gly71Arg), rs2306283 (Asn130Asp/N130D, A388G/388A>G), and rs4149056 (c.521T>C, Val174Ala, or V174A) were genotyped using the Sequenom® MassARRAY® and iPLEX™ Gold genotyping assay (Sequenom Laboratories; San Diego, CA, USA). Briefly, the three SNP loci were subjected to PCR assay with the designed primers (Table 1) in a 5 *μ*l of the reaction system containing 4 *μ*l of PCR Master Mix (Thermo Fisher Scientific, Inc.; Waltham, MA, USA) and 1 *μ*l of DNA template under the following conditions: at 94°C for 5 min; followed by 45 cycles of at 94°C for 20 s, at 56°C for 30 s, and at 72°C for 1 min; and finally at 72°C for 3 min. The PCR products were treated with shrimp alkaline phosphatase (SAP; Thermo Fisher Scientific, Inc.; Waltham, MA, USA) to remove the free dNTPs. Then, a single-base extension reaction was performed with the primers (Table 1) under the following conditions: at 94°C for 30 s; followed by 40 cycles of at 94°C for 5 s and 5 cycles of at 52°C for 5 s, and at 80°C for 5 s; and finally at 72°C for 3 min. The extension products were purified with resin, and then subjected to matrix-assisted laser desorption/ionization time of flight mass spectrometry (MALDI-TOF). The MALDI-TOF data were processed using the software TYPER version 4.0.

The rs8175347 locus was genotyped using direct sequencing. Briefly, PCR assay was performed in a 25 *μ*l of the reaction system containing 12.5 *μ*l of KAPA 2G Robust Mix (KAPABIOSYSTEMS, Woburn, MA, USA), 1 *μ*l of each forward and reverser primers (10 mmol/L; Table 1), 1 *μ*l of DNA template, and 9.5 *μ*l of ddH_2_O under the following conditions: at 95°C for 5 min; followed by 30 cycles of at 95°C for 30 s, at 57.5°C for 30 s, and at 72°C for 1 min; and finally at 72°C for 5 min. Following purification, the PCR products were sequenced on an ABI 3730xl DNA Sequencer (Applied Biosystems; Foster City, CA, USA), and the sequence data were analyzed with the software Mutation Surveyor version 4.0.6 (SoftGenetics; State College, PA, USA).

The rs1805173 locus was genotyped using the PCR-short tandem repeat (STR) analysis. Briefly, PCR assay was performed in a 25 *μ*l of the reaction system containing 12.5 *μ*l of KAPA 2G Robust Mix, 1 *μ*l of each forward and reverser primers (10 mmol/L; Table 1), 1 *μ*l of DNA template, and 9.5 *μ*l of ddH_2_O under the following conditions: at 95°C for 5 min; followed by 30 cycles of at 95°C for 30 s, at 57°C for 30 s, and at 72°C for 1 min; and finally at 72°C for 5 min. PCR products were analyzed using an ABI3730XL capillary electrophoresis platform(Applied Biosystems; Foster City, CA, USA) according to the manufacturer's instructions. Allele sizes were scored using GeneMapper® 4.0 software package (Thermo Fisher Scientific; Foster City, CA, USA).

Dinucleotide (GT)_n_ repeats were assigned into three groups according to the* n* value,* n *< 24, short repeats;* n* = 24 to 29, medium repeats;* n *> 29, long repeats.

### 2.6. Statistical Analyses

All measurement data were described as mean ± standard deviation (SD), and all categorical data were expressed as proportions. Differences of means were tested for statistical significance with independent-samples* T* test, and comparisons of categorical data were done with chi-square test. The Hardy-Weinberg equilibrium was tested and the genotype and allele frequencies were compared with chi-square test. The variables with statistical significance were included in the multivariate conditional logistic regression model. All statistical analyses were conducted with the statistical software SPSS version 17.0 (SPSS, Inc.; Chicago, IL, USA), and a* P *value< 0.05 was considered statistically significant.

## 3. Results

### 3.1. Demographic and Clinical Characteristics of the Study Subjects

There were no significant differences detected between the case and control groups in terms of gender (*P* = 0.889), body weight (*P* = 0.857), gestational age (*P* = 0.51), days of age (*P* = 0.709), types of delivery (*P* = 0.596), or types of feeding (*P* = 0.19); however, the serum TBIL (*P* = 0), UCB (*P* = 0), and DBIL levels (*P* = 0) were higher in the case group than in the control group (Table 2).

### 3.2. Incidence of ABO Incompatibility and G6PD Deficiency

Significantly higher incidence of ABO incompatibility (21% versus 9.6%,* P* = 0) and G6PD deficiency (7% versus 2%,* P* = 0) was detected in the case group compared to the control group (Table 2).

### 3.3. Genotype Frequency of UGT1A1, OAP1B1, and HO-1 Genes

Except the rs4149056 locus, the distributions of rs2306283 (*P*_H-W_ = 0.533), rs4148323 (*P*_H-W_ = 0.957), rs8175347 (*P*_H-W_ = 0.194), and rs1805173 genotypes (*P*_H-W_ = 0.091) were all in accordance with the Hardy-Weinberg equilibrium (Table 3). No TA_7_/TA_7_ homozygous mutation was detected at the rs8175347 locus, and the number of GT repeats ranged from 16 to 37 at the rs1805173 locus. There were significant differences in the frequencies of rs4148323 (*P* = 0) and rs1805173 genotypes (*P* = 0.017) between the case and control groups, and no significant differences were found between groups in terms of the frequency of rs8175347 (*P* = 0.197) or rs2306283 genotypes (*P* = 0.239). At the rs4148323 locus, the frequencies of GA heterozygotes and AA mutant homozygotes were higher in the case group than in the control group (GA, 38.1% versus 31.6%,* P* = 0.02; AA, 10.8% versus 3.2%,* P* = 0), and at the rs1805173 locus, the frequencies of LS, MS, and SS genotypes were all significantly higher in the case group than in the control group (LS, 29.4% versus 24.8%,* P* = 0.011; MS, 17.5% versus 14.4%,* P* = 0.022; SS, 10.1% versus 4.8%,* P* = 0.001) (Table 3).

### 3.4. Allele Frequency of UGT1A1, OAP1B1, and HO-1 Genes

There were significant differences in the frequencies of rs4148323 (*P* = 0) and rs1805173 alleles (*P* = 0.013) between the case and control groups (*P *< 0.05), and no significant differences were found between groups in terms of the frequency of rs8175347 (*P* = 0.22) or rs2306283 alleles (*P* = 0.992). A higher frequency of rs4148323 A allele was detected in the case group than in the control group (29.9% versus 19%,* P* = 0), and a higher frequency of rs1805173 S allele was detected in the case group than in the control group (33.6% versus 24.4%,* P* = 0) (Table 4).

### 3.5. Genetic Risk Factors of Neonatal Hyperbilirubinemia

All variables with statistical significance detected by univariate analysis were included in the multivariate conditional logistic regression model, and multivariate logistic regression analysis revealed that the mutant genotype of rs4148323 in the UGT1A1 gene, ABO incompatibility, G6PD deficiency, and SS genotype at rs1805173 locus of the HO-1 gene were genetic risk factors of neonatal hyperbilirubinemia (Table 5).

## 4. Discussion

Neonatal hyperbilirubinemia is a global pediatric concern [2]. Although the exact causes of hyperbilirubinemia remain unclear [6], the contribution of genetic factors to the pathogenesis of hyperbilirubinemia has been paid more and more attention [24]. To date, there is no knowledge on the genetic risk factors of neonatal hyperbilirubinemia in Fujian Province, Southeastern China. This case-control study was therefore designed to examine the associations of ABO incompatibility, G6PD deficiency, and the polymorphisms of the UGT1A1, OATP1B1, and HO-1 genes with neonatal hyperbilirubinemia in Southeastern China, and the results of multivariate logistic regression analyses showed that the mutant genotype of rs4148323 in the UGT1A1 gene, ABO incompatibility, G6PD deficiency, and the SS genotype of the (GT)_n_ repeats in the promoter region of the HO-1 gene were genetic risk factors for neonatal hyperbilirubinemia in Southeastern China.

The associations between the GT repeats in the promoter region of the HO-1 gene and risk of neonatal hyperbilirubinemia are reported to vary in regions and populations. In a prospective case-control study to assess the association between HO-1 gene variants and hyperbilirubinemia risk in Indian newborns, the incidence of short (GT)_n_ allele (≤ 20) was three times higher in hyperbilirubinemic neonates than in controls, and short (GT)_n_ repeats of HO-1 gene were identified as an independent risk factor for neonatal hyperbilirubinemia (*OR* = 4.4, 95%* CI* = 1.2–16.8) [14]. Results from a prospective study including 444 healthy infants born in Taiwan from 2013 to 2015 showed a higher frequency of short HO-1 promoter GT-allele (<24 repeats) in hyperbilirubinemic infants than in nonhyperbilirubinemic infants (*P *< 0.05), and short HO-1 promoter GT-repeat was associated with an increased risk of neonatal hyperbilirubinemia (*RR* = 2.185; 95%* CI* = 1.527–3.125) [25]. However, no association was detected between the short HO-1 promoter GT-repeat and hyperbilirubinemia risk in Turkish neonates [26]. In this study, the number of GT repeats was found to range from 16 to 37, and the frequencies of LS, MS, and SS genotypes and S allele were significantly higher in the case group than in the control group (*P *< 0.05). Multivariate logistic regression analysis revealed that the SS genotype of the GT repeats in the HO-1 gene was a risk factor for neonatal hyperbilirubinemia in Southeastern China (*OR* = 3.051, 95%* CI* = 1.417–6.57). Our findings were in agreement with the results from the study conducted in Taiwan [25], which may be attributed to the fact that Fujian and Taiwan are geographically proximal. It is therefore hypothesized that the short HO-1 promoter GT-repeat is associated with the risk of neonatal hyperbilirubinemia in Fujian, Southeastern China.

Currently, the correlation between UGT1A1 polymorphisms and risk of neonatal hyperbilirubinemia mainly focuses on the TATA box in the promoter region and the coding region [27, 28]. It was reported that the coding region mutation of the UGT1A1 gene was highly prevalent in Asia, with G211A as the predominant type of mutations [29, 30]. In this study, the frequency of the A allele mutation at G211A variant was significantly higher in the case group than in the control group, which was similar to the that detected in Guangdong (28.7%) [11], Taiwan [25], and was higher than that in Guangxi (20.4%) [31]. This mutation leads to mutation of GGA to AGA at codons 71, and transformation of the corresponding coding amino acids from glycine to arginine, thereby affecting the enzyme functions [32]. In addition, our findings indicated that the G211A variant of the UGT1A1 gene was a risk factor for neonatal hyperbilirubinemia in Fujian, Southeastern China, and the AA homozygous mutant newborns had a higher risk of hyperbilirubinemia than heterozygous mutant newborns. These data are inconsistent with previous studies conducted in Taiwanese neonates concluding that the carriage of the homozygous 211 G to A variation within the coding region in the UGT1A1 gene is a risk factor for neonatal hyperbilirubinemia [25, 33, 34]. Currently, the associations of the TATA box in the promoter region of the UGT1A1 gene with the risk of neonatal hyperbilirubinemia are reported to vary greatly in populations and regions [35]. The correlation between (TA)_7_ insert sequence and the risk of neonatal hyperbilirubinemia has been widely detected in Caucasians in western countries, while no apparent associations were seen between the T genotype polymorphisms in this region and risk of neonatal hyperbilirubinemia in Asian populations [28, 29]. Moreover, (TA)_7_ mutation was found to be protective during the progression of neonatal hyperbilirubinemia [11], which was completely contrary to previous reports [28, 29]. In the current study, we did not detect a (TA)_7_ homozygous insertion mutation, and no significant difference was found in the frequency of the genotype between the case and control groups (*P *> 0.05).

To date, the associations of OATP1B1 polymorphisms with the risk of neonatal hyperbilirubinemia mainly focus on G388A and T521C variants [11, 19, 36]. G388A mutation leads to the transformation of the amino acid encoded by codons 130 of the OATP1B1 from asparagine to aspartic acid, resulting in a reduction in the ability to eliminate UCB [37]. T521C mutation is reported to cause a rise in the plasma bilirubin level and is associated with neonatal jaundice [38]. Previous studies have shown diverse associations between the OATP1B1 polymorphisms and neonatal hyperbilirubinemia in various regions of China. G388A mutation was found to correlate with neonatal hyperbilirubinemia in Guangdong [11], and A388G mutation was identified as a risk factor for severe neonatal hyperbilirubinemia in northern China [39]. In addition, the polymorphisms in the T521C variant, but not G388A, were strongly associated with neonatal hyperbilirubinemia in Yunnan [40], and G388A and T521C variants showed no associations with the risk of neonatal hyperbilirubinemia in Guangxi, and various genotypes had few effects on serum bilirubin levels [41]. In the present study, the distribution of the T521C variant did not pass the Hardy-Weinberg equilibrium test; therefore, we failed to examine the associations of T521C mutation with neonatal hyperbilirubinemia in Fujian, Southeastern China, while the polymorphisms in the G388A variant had no associations with neonatal hyperbilirubinemia in Fujian, Southeastern China, which was similar to previous findings reported in Taiwan [25].

In the current study, the incidence of ABO incompatibility and G6PD deficiency was 2.1 and 3.5 times greater in the case group than in the control group, and multivariate conditional logistic regression analysis identified both ABO incompatibility and G6PD deficiency to be risk factors for neonatal hyperbilirubinemia in Southeastern China, which was in agreement with previous reports [42, 43]. If the mother-child ABO blood types are incompatible, the maternal anti-ABO blood type antibody may bind to the corresponding antigen on fetal red blood cell membrane surface to induce hemocatheresis and hemolysis, resulting in jaundice and anemia [44]. G6PD deficiency, an X-linked incomplete dominant hereditary disease, is caused by deficiency of the enzyme G6PD due to G6PD gene mutations [18]. It was reported that the overall prevalence of G6PD deficiency was 1.37% in Fujian Province [45]. In addition to hyperbilirubinemia, the risk factors of bilirubin encephalopathy include combined isoimmune hemolysis, G6PD deficiency, apnea, septicemia, metabolic acidosis, and hypoalbuminemia [3, 5, 46]. A recent meta-analysis to identify the risk factors of severe neonatal hyperbilirubinemia in low- and middle-income countries showed that ABO incompatibility and G6PD deficiency led to a clear-cut increase in the risk of severe hyperbilirubinemia and bilirubin encephalopathy in hyperbilirubinemic newborns [12]. In addition, the combined presence of ABO incompatibility and G6PD deficiency may lead to the development of neonatal jaundice ahead of time [47].

In summary, the results of the present study demonstrate that G211 mutation in the UGT1A1 gene, ABO incompatibility, G6PD deficiency, and the SS genotype of the (GT)_n_ repeats in the promoter region of the HO-1 gene are risk factors for neonatal hyperbilirubinemia in Fujian, Southeastern China, which provides insights into the elucidation of the contribution of genetic factors to pathogenesis of hyperbilirubinemia and the management of hyperbilirubinemia. Notably, hyperbilirubinemia caused by UGT1A1 gene polymorphisms and locus-specific mutations in the HO-1 gene should be considered during the diagnosis and treatment of hyperbilirubinemic infants with unknown causes.

## Figures and Tables

**Table 1:** Sequences of PCR primers and single-base extension primers for 5 loci in three genes.

Gene	Variant	Sequence of PCR primers (5′-3′)	Sequences of single-base extension primers
OAP1B1	rs2306283	F: ACGTTGGATGGATGTTCTTACAGTTACAGG; R: ACGTTGGATGACAAGTGGATAAGGTCGATG	CGATGTTGAATTTTCTGATGAAT
	rs4149056	F: ACGTTGGATGAATCTGGGTCATACATGTGG; R:ACGTTGGATGCCAATGGTACTATGGGAGTC	CCAAGCATATTACCCATGAAC
UGT1A1	rs4148323	F: ACGTTGGATGCTGACGCCTCGTTGTACATC; R:ACGTTGGATGCACATCCTCCCTTTGGAATG	GACTTCTTCAAGGTGTAAAATGCTC
	rs8175347	F: AACTCCCTGCTACCTTTGTGG; R:ATGGCACAGGGTACGTCTTC	-
HO-1	rs1805173	F: AGAGCCTGCAGCTTCTCAGA; R:ACAAAGTCTGGCCATAGGAC	-

**Table 2:** Comparison of the demographic and clinical features between the case and control groups.

Characteristics		Case group (*n* = 286)	Control group (*n *= 250)	*P* value
Gender	Males	179	155	0.889
	Females	107	95	
Body weight (g)		3187±471.9	3180±353.2	0.857
Gestational age (weeks)		38.42±1.738	38.33±1.474	0.51
Days of age (d)		5.808±4.338	5.948±4.353	0.709
Type of delivery	Vaginal delivery	178	150	0.596
	Caesarean delivery	108	100	
Type of feeding	Breastfeeding	107	80	0.19
	Others	179	170	
TBIL (*μ*mol/L)		339.2±65.94	72.94±48.94	0
UCB (*μ*mol/L)		325.5±64.59	66.51±48.26	0
DBIL (*μ*mol/L)		13.73±12.48	6.458±7.953	0
No. cases with ABO incompatibility		60	24	0
No. cases with G6PD deficiency		20	5	0.006

**Table 3:** Comparison of genotype frequencies in each SNP locus of the UGT1A1, OAP1B1, and HO-1 genes between the case and control groups.

Gene	SNP locus		Frequency in the case group(%)	Frequency in the control group (%)	*P*_H-W_	*χ* ^2^	*P*	*OR(*95%*CI)*
UGT1A1	(TA)n repeat rs8175347	TA_6_/TA_6_	84.3	80	0.194	1.665	0.197	1
		TA_6_/TA_7_	15.7	20				1.339(0.859–2.088)
		TA_7_/TA_7_	0	0				-
	rs4148323	GG	51	65.2	0.957	16.95	0	1
		GA	38.1	31.6				1.504(1.069–2.221)
		AA	10.8	3.2				4.326(1.927–9.712)
OAP1B1	rs2306283	GG	55.6	58.4	0.533	2.863	0.239	1
		GA	39.2	33.6				1.224(0.853–1.757)
		AA	5.2	8				0.689(0.340–1.395)
	rs4149056	TT	80.4	84.4	0.002			
		TC	11.9	10.4				
		CC	7.7	5.2				
HO-1	(GT)n repeat rs1805173	LL	22.4	34.4	0.091	13.84	0.017	1
		LM	14.3	14.4				1.530 (0.881–2.659)
		LS	29.4	24.8				1.821(1.148–2.886)
		MM	6.3	7.2				1.344(0.648–2.786)
		MS	17.5	14.4				1.866(1.091–3.193)
		SS	10.1	4.8				3.247(1.539–6.851)

**Table 4:** Comparison of frequencies of the alleles in each SNP locus of the UGT1A1, OAP1B1, and HO-1 genes between the case and control groups.

Gene	SNP locus	Allele	Frequency in the case group(%)	Frequency in the control group (%)	*χ* ^2^	*P*	*OR(*95%*CI*)
UGT1A1	(TA)n repeat rs8175347	TA_6_	92.1	90	1.503	0.22	1
		TA_7_	7.9	10			1.301 (0.853–1.984)
	rs4148323	G	70.1	81	16.98	0	1
		A	29.9	19			1.818 (1.365–2.421)
OAP1B1	rs2306283	G	75.2	75.2	0	0.992	1
		A	24.8	24.8			1.001 (0.758–1.322)
HO-1	(GT)n repeat rs1805173	L	44.2	54	6.114	0.013	1
		M	22.2	21.6			1.255 (0.922–1.709)
		S	33.6	24.4			1.680 (1.264–2.232)

**Table 5:** Logistic regression analysis of genetic risk factors for neonatal hyperbilirubinemia.

Risk factor		*β*	SE	Wald*χ*^2^	*P*	*OR(*95%*CI*)
SNP rs4148323 in the UGT1A1 gene*∗*	AA	1.459	0.426	11.76	0.001	4.303 (1.869–9.911)
	GA	0.429	0.194	4.877	0.027	1.536(1.049–2.248)
G6PD deficiency		1.355	0.522	6.733	0.009	3.877(1.393–10.79)
ABO incompatibility		0.971	0.267	12.22	0	2.64(1.564–4.455)
(GT)n repeat rs1805173 in the HO-1 gene^#^	LM	0.437	0.292	2.233	0.135	1.548(0.873–2.744)
	LS	0.46	0.246	3.504	0.061	1.585(0.979–2.566)
	MM	0.449	0.382	1.379	0.24	1.567(0.74–3.316)
	MS	0.443	0.289	2.357	0.125	1.557(0.885–2.742)
	SS	1.116	0.391	8.125	0.004	3.051(1.417–6.57)

*∗* The wild-type GG genotype served as a reference for SNP rs4148323 in the UGT1A1 gene; # the wild-type GG genotype served as a reference for (GT)n repeat variant rs1805173 in the HO-1 gene.

## Data Availability

The data used to support the findings of this study are available from the corresponding author upon request.
